# Early clinical evaluation of the Hugo robotic-assisted surgery (RAS) for performing radical prostatectomy: an IDEAL stage 2 study

**DOI:** 10.1136/bmjsit-2024-000360

**Published:** 2025-04-05

**Authors:** Andrew Shepherd, Ata Jaffer, Angus Bruce, Daniel Chia, Prokar Dasgupta, Ben Challacombe

**Affiliations:** 1 Urology, Guy's and St Thomas’ NHS Foundation Trust, London, UK; 2 The University of Adelaide Adelaide Medical School, Adelaide, South Australia, Australia; 3 King’s College London, London, UK

**Keywords:** Development Study, Urology Devices, Robotic Surgical Procedures, Minimally Invasive Surgical Procedures

## Abstract

**Objectives:**

To assess the feasibility and safety of the new Hugo robotic-assisted surgery (RAS) system for robotic-assisted radical prostatectomy (RARP), describing iterative changes in our operative technique—IDEAL stage 2.

**Design:**

Prospective, single-centre series.

**Setting:**

Tertiary urological unit in London, UK.

**Participants:**

Male patients diagnosed with clinically localised prostate cancer and suitable for RARP from February 2023 to May 2024.

**Main outcome measures:**

The primary outcome was to assess the safety of using the device without converting to the existing robotic platform (da Vinci), laparoscopy or open. Secondary outcomes assessed surgical (operative time, blood loss, time to catheter removal, complications), oncologic (surgical pathology and margin status) and early functional (continence) outcomes.

**Results:**

50 patients were included in the study. No cases required conversion to an existing robotic platform, laparoscopy or open, and there were no intraoperative surgical complications. Mean age was 60 years and mean prostate-specific antigen was 12.2 ng/mL. The mean operative time was 148 min and estimated blood loss was 168 mL. Mean length of stay was 1.5 days and mean length of catheter duration was 13 days. On final pathology, 18 patients (36%) had T3 disease and four had positive surgical margins (8%). The mean International Consultation on Incontinence Questionnaire–Urinary Incontinence score for urinary continence at 3 months was 7. There were six Clavien-Dindo grade 2 complications and two Clavien-Dindo 3a complications. There were four instances of recoverable, temporary device failure. Iterative improvements were made to docking setup, use of robotic instruments and reduction in robotic arm collisions.

**Conclusions:**

We demonstrated feasibility and the safe introduction of the Hugo RAS for RARP into an experienced robotic urological programme. Perioperative, early oncological and functional outcomes were similar to other early series. Further studies will aim to describe the learning curve with this robot and optimisation of surgical quality.

**WHAT IS ALREADY KNOWN ON THIS TOPIC**

- The new Hugo robotic-assisted surgery (RAS) system for radical prostatectomy is in its infancy with small series reporting early outcomes showing its promise for this surgical indication.

**WHAT THIS STUDY ADDS**

- This study describes modifications in technique during the implementation of the new Hugo RAS, demonstrating its safety and feasibility for radical prostatectomy.

**HOW THIS STUDY MIGHT AFFECT RESEARCH, PRACTICE OR POLICY**

- This study demonstrates the safe introduction of the Hugo RAS for radical prostatectomy, allowing wider use of this new platform.

## Introduction

 Robotic-assisted surgery (RAS) for the treatment of clinically localised prostate cancer has long been established but dominated by a single manufacturer. With the expiration of patents on the Intuitive Surgical da Vinci robotic system (Intuitive Surgical, Sunnyvale, USA) in 2019, the marketplace has rapidly expanded with new platforms now commercially available.^1^ The Medtronic Hugo RAS system (Medtronic, Minneapolis, USA) is now available in Europe, approved for all major benign and malignant urological procedures. The Hugo RAS system is notable for its modular design with an open console and the ecosystem which integrates advanced robotic technology, artificial intelligence and state-of-the-art imaging capabilities.^1^ Robotic-assisted radical prostatectomy (RARP), the most frequently performed RAS in urology, was first performed with a Hugo robot in June 2021.^2^

We describe the introduction of the Hugo RAS system for RARP in the UK at our high-volume robotic centre, presenting our evaluation according to the principles described by the IDEAL Collaboration.^3^ The focus of our evaluation was the safe introduction of the Hugo RAS system, exploring its feasibility and refinements in our technique in our early cases—stage 2 of the IDEAL framework.

## Methods

### Preparation and prior experience

The Hugo RAS was introduced into an existing high-volume robotic urological service, where over 350 RARPs are performed annually. Our unit has extensive robotic urological experience covering the gamut of indications including both benign and malignant conditions, upper and lower urinary tract procedures. Two experienced surgeons (each >1000 RARP cases prior to study commencement) and their respective teams took part in extensive dry and wet lab training before undertaking the procedures. Remote mentoring using Proximie (Proximie, London, UK) was used for the initial cases. Proximie is a mixed reality system which allows transmission of audio and video across sites. The mentor can guide the mentee by annotation on the video feed, by demonstrating anatomic landmarks, for example, as well as providing voice instructions without the need to travel to the host institution. It was provided to the study gratis as part of an internal collaboration with King’s Health Partners as the Proximie CEO is a surgical colleague at our institution. The first two cases in our series were mentored by audio and video by a Belgian urologist. The operating surgeons also continued to use the da Vinci robotic system for cases during the study period. Patients and the public were not involved in the design or conduct of this study.

### Case selection

Having learnt from the experience of the introduction of other novel surgical platforms into our robotic urological programme,^4^ we carefully selected the initial cases in our series to allow the surgeon to focus on the safe utilisation of the new robotic system. We used the same theatre suite, operating room team, anaesthetic team and thus in the early cases, the only change to our usual procedure was the introduction of the Hugo RAS. In the initial 10 cases, patients with intermediate grade prostate cancer (International Society of Urological Pathology (ISUP) grade group 2), organ confined disease (cT2), body mass index <30 kg/m^2^, prostate volume <50 cm^3^ and American Society of Anesthesiologists Classification ≤2 were selected. Once we demonstrated the ability to safely complete the first 10 cases, there were no restrictive case selection criteria.

### Operative details

Each case was performed via a transperitoneal, anterior approach with the patient placed supine in Trendelenburg position and placement of six robotic ports via an open Hasson technique. A posterior reconstruction was performed and the vesicourethral anastomosis performed in a running, continuous fashion, as described by Van Velthoven *et al*.^5^ Routine catheter removal was performed after 7–14 days in our institution.

### Outcomes

The primary outcome was to assess the safety of using the device without converting to the existing robotic platform (da Vinci), laparoscopy or open. Secondary outcomes assessed surgical (operative time, blood loss, time to catheter removal, complications), oncologic (surgical pathology and margin status) and early functional (continence) outcomes. Patients were followed up at 8 weeks and 3 months postoperatively; erectile function and biochemical recurrence rates were not analysed, given the short follow-up in this study.

## Results

A total of 50 patients underwent RARP using the Hugo RAS system during the study period from February 2023 to May 2024. All patients completed at least 3 months of postoperative follow-up. No patients in the study underwent pelvic lymphadenectomy.

### Baseline characteristics

The mean age of patients in the cohort was 60 years, ranging from 50 to 73 years. The mean body mass index was 28 kg/m^2^, ranging from 21 to 36 kg/m^2^. The majority of patients (84%) had intermediate grade prostate cancer (ISUP grade group 2 or 3) and clinically 80% had organ-confined disease (clinical stage 1 or 2). The full outline of baseline characteristics can be seen in table 1.

**Table 1:** Patient demographics and preoperative pathological characteristics

	Mean (SD)
Age, years	60 (6)
Body mass index, kg/m^2^	28 (4)
Prostate-specific antigen, ng/mL	12.2 (10.6)
Prostate volume on MRI, cm^3^	41 (24)
ISUP grade group, n (%)	
1	0 (0)
2	27 (54)
3	16 (32)
4	5 (10)
5	2 (4)
T stage, n (%)	
1–2	40 (80)
3	10 (20)

ISUPInternational Society of Urological Pathology

### Primary outcome

No cases required conversion to an existing robotic platform (da Vinci), laparoscopy or open.

### Secondary outcomes

A summary of the intraoperative and early postoperative outcomes is outlined in table 2. The mean operative time was 148 min, ranging from 90 to 230 min. Estimated blood loss on average was 168 mL, with a range of 50–650 mL. There were no intraoperative surgical complications encountered. Patients stayed on average for 1.5 days as an inpatient and had their urethral catheter removed after 13 days on average. There were six Clavien-Dindo 2 complications in six different patients^6^:

- Ileus
- Readmission with constipation.
- Two readmissions with urinary tract infection.
- Two vesicourethral anastomotic leaks seen on planned cystogram (performed when there was a large bladder neck reconstruction).

**Table 2:** Perioperative results

	Mean (SD)
Operative time, minutes	148 (32)
Estimated blood loss, mL	168 (120)
Intraoperative complications	0
Device failures	3 recoverable arm failures, 1 monitor failure
Inpatient length of stay, days	1.5 (1)
Time to trial without catheter, days	13 (6)
30-day complications	8: 6 Clavien-Dindo 2, 2 Clavien-Dindo 3a

There were two Clavien-Dindo 3a complications in two patients.

- Vesicourethral anastomotic leak requiring percutaneous pelvic drain insertion for urinoma drainage.
- Vesicourethral anastomotic leak following trial without catheter requiring flexible cystoscopic-guided replacement of the urethral catheter.

**Figure 1:**
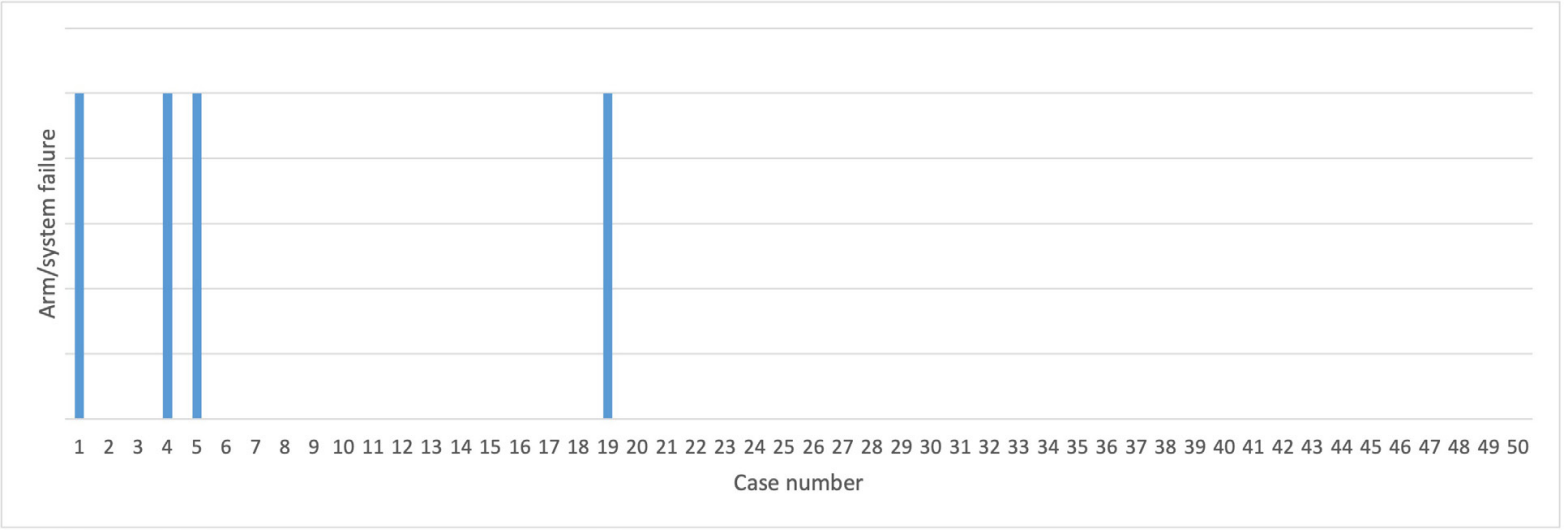
Incidence of temporary device failures, shown by consecutive case number.

There were four intraoperative device-related failures, all recoverable with a 10 min reboot of the affected component. Three were temporary arm failures and one was a monitor failure. All of these component failures occurred within the first 20 cases of the series (see figure 1).

The pathological and functional outcomes are summarized in table 3. Pathologically, 98% of cases had intermediate grade disease (ISUP grade group 2 or 3) on their final pathology result, with a single case of ISUP grade group 5 disease. There were four positive margins in the series, 8% overall. Continence was assessed at 3 months using the International Consultation on Incontinence Questionnaire–Urinary Incontinence assessment, a validated instrument.^7^ Scores range from 0 to 21, with a lower score indicating a better continence outcome.

**Table 3:** Surgical pathology/postoperative results

	n (%)
ISUP grade group	
1	0 (0)
2	29 (58)
3	20 (40)
4	0
5	1 (2)
T stage	
2	32 (64)
3a	14 (28)
3b	4 (8)
Positive surgical margins	4 (8)
ICIQ-UI at 3 months, mean	7

ICIQ-UIInternational Consultation on Incontinence Questionnaire–Urinary IncontinenceISUPInternational Society of Urological Pathology

### Evolution of operative approach/iterative changes

We made several iterative changes during our development phase, using the instruments of the Hugo RAS to accomplish the requisite tissue handling expected as well as experimentation with the docking angles to settle on a preferred docking approach.

#### Docking angles and docking sequence

The modular nature of the Hugo RAS allows for flexibility in the docking positions of the robotic arms; Medtronic provided a recommendation on the docking angles for each of the arms for an RARP.^8^ After initially rigidly adhering to these docking angle recommendations, we realised that there was some flexibility in these angles without compromising the operative success or causing any adverse impact on intraoperative arm collisions. Seemingly, a 5 degree margin of error (either way of the recommended angle) was tolerable, allowing for a faster docking time and tailoring according to patient anatomy. The docking time was shortened with case experience and further improved once we optimised the sequence of docking the arms.

#### Tissue grasping forceps

One of the difficulties encountered was the lack of a strong tissue grasping forceps to be used with the fourth robotic arm that would be analogous to the Prograsp forceps (Intuitive Surgical) with which we were familiar with the da Vinci system. The Medtronic Cadiere forceps developed for this purpose were short with poor gripping strength, requiring frequent regrasping of tissues and prolonging operative time. We repurposed a toothed grasping forceps with success as well as the extra large needle holding forceps to accomplish the same tissue holding capability that we would have expected. This shortcoming was particularly noticeable during the seminal vesicle dissection and nerve-sparing steps where precise traction with the fourth arm is advantageous to completing the step efficiently and safely. With further iterations of the Hugo RAS, Medtronic is developing a more suitable tissue grasping forceps, removing the need to repurpose other instruments.

#### Robotic alarming and instrument collisions

Early in the series, we were hampered by interruptions to the case flow due to frequent alarming of the RAS and freezing of the movement of the instruments until the error was acknowledged; this required the operative surgeon to click on the console touch pad to acknowledge the error before the motion of the robotic arms was restored. Often, this alarming situation was due to the close proximity of the robotic arms or gentle instrument clash visible within the endoscopic view of the camera. After a software update of the RAS (approximately at case 30 in our series), the frequency of the alarming significantly reduced, improving case flow. After the software update, there were also no further instances of device failures, which all occurred within the first 20 cases of our series.

## Discussion

We have shown that the introduction of the Hugo RAS into an existing robotic centre can allow RARP to be performed safely without any conversions or compromise of early oncological outcomes. Our perioperative results compare favourably with other published series with similar operative time, estimated blood loss and lack of conversions.^29^,^12^ Our complication rates and early oncological outcomes also are in keeping with other published series evaluating the Hugo RAS for RARP,^29^,^11^ even though we had higher rates of pathologically locally advanced (pT3) disease in comparison (36% in our series compared with 31% in Bravi *et al*).^10^

The four recoverable, device-related failures occurred early in the series. We did not encounter any further instances after the 19th case in our series, which seemed to coincide with the timely software upgrade which also addressed the frequent issues with instrument alarming. The relaxation of adherence to the docking angles for the robotic arms may have also contributed to this reduction as we were able to tailor our docking to patient anatomy and reduce instrument collisions by ensuring adequate spacing between the instruments.

Although we did not formally assess the docking times for our cases, anecdotally this improved dramatically once we were through the initial 10 or so cases—familiarity with the equipment certainly playing a major part in this improvement. We observed further improvement in this aspect after experimentation with docking angles, reducing the time spent ensuring the precise docking angle was completed when mounting to the laparoscopic port.

The absence of a grasping instrument, similar to the Prograsp forceps (Intuitive Surgical) we use with the da Vinci system for RARP, is an area for ongoing improvement with the Hugo RAS. We understand that Medtronic will be addressing this shortcoming in an imminent hardware update to the Hugo RAS, along with the introduction of integrated stapling and energy devices. The use of alternative instruments such as the toothed grasping forceps and the extra-large needle driver helped to overcome this deficiency.

After the 20th case in the series, we only had two Clavien-Dindo complications (both grade 2) and no further device-related failures, demonstrating stability in the safety of the system and familiarity with the device. We have been able to reliably reproduce the docking and setup in a timely fashion and complete the cases promptly without undue delays owing to device alarming that were evident in the early cases in the series.

The open console design has allowed for more free communication with the operative theatre team, including the bedside assistant. The ability to observe the entire operative team with the open console also improved the dynamics between the members of the theatre team.

From the surgeon’s perspective, we have also observed some key differences to the da Vinci. There is a slight pause when switching energy from monopolar to bipolar, and the sound made when activating both forms of energy is the same. The laparoscope is automatically flipped upside down by the robot when inserted into the patient, regardless of the orientation when it is docked, so requires manual rotation either by the bedside assistant or the surgeon. There is no ability for dual console compatibility at present, potentially impacting the teaching benefits of the Hugo. From the assistant’s perspective, there is the potential for harmful clashes with the assistant from the robotic arms due to their wide motion and thus a keen sense of position and attention is required to avoid injury. The surgeons continued to perform da Vinci RARP concurrently during the study period, which did not seem to adversely affect the outcomes of using the Hugo RAS. As a teaching institution, we continued our fellowship training programme during the introduction of the Hugo RAS and allowed our fellows to train during these cases as we do on the da Vinci.

### Limitations

Although the data in the series are prospective, the follow-up is short term and we have thus not reported on our erectile function outcomes. We await the maturation of our cohort to report our longer-term continence and erectile functional outcomes and oncological outcomes. We believe that after our first 50 cases we have demonstrated stability with our technique and outcomes; further improvements will occur with new hardware in due course from Medtronic.

## Conclusions

We have demonstrated the feasibility and safe introduction of the Hugo RAS for RARP into an experienced robotic urological programme. Perioperative, early oncological and functional outcomes are in keeping with other Hugo RAS RARP series. With alterations to our technique in this initial series, we have reached a level of stability in our outcomes and look to proceed to the next IDEAL stage to evaluate the learning curve with this device, optimise surgical quality and aim to ultimately proceed to a multicentre, randomised controlled trial (IDEAL stage 3).

## Data Availability

Data are available on reasonable request.
